# Revolutionizing pharmacokinetics: the dawn of AI-powered analysis

**DOI:** 10.3389/jpps.2024.12671

**Published:** 2024-02-16

**Authors:** Ali Ghayoor, Hamed Gilzad Kohan

**Affiliations:** College of Pharmacy, Western New England University, Springfield, MA, United States

**Keywords:** pharmacokinetics, artificial intelligence, machine learning, pharmacokinetic prediction, computational pharmacology

## Abstract

This editorial explores how artificial intelligence (AI) is revolutionizing the science of pharmacokinetics (PK). It discusses the challenges of conventional PK analysis and how AI has transformed this area. It highlights the promise of artificial intelligence (AI) in predicting pharmacokinetic profiles from chemical structures and its application in several aspects of pharmacology, including dosage customization and drug interactions. Additionally, it emphasizes how important ethical issues and openness are to AI applications, especially when it comes to pharmacokinetic prediction and dataset adaptation. Future directions for AI in PK are discussed, with the creation of all-inclusive AI pharmacokinetics/pharmacometrics software being envisioned. Drug discovery and patient care could be transformed toward more individualized and effective healthcare solutions with the help of this software, which could handle tasks such as data cleaning, model selection, and regulatory report preparation. The editorial highlights the importance of AI in improving pharmaceutical sciences while urging caution and teamwork in navigating its possible uses in pharmacokinetics.

“The computer is incredibly fast, accurate, and stupid. Man is incredibly slow, inaccurate, and brilliant. The marriage of the two is a force beyond calculation.”

- Leo Cherne.

## Introduction

Pharmacokinetics (PK) plays a vital role in drug development, offering insights crucial for dosing and efficacy. Yet, conventional PK analysis is laden with challenges, often being a complex and labor-intensive process that necessitates extensive data collection and processing. In recent years, the integration of Artificial Intelligence (AI) has heralded a paradigm shift, offering solutions to these enduring challenges. Olga Obrezanova’s work [1] delineates how AI has emerged as an indispensable tool for simulating *in vivo* PK in animals and humans. Leveraging machine learning and generative AI techniques, we are now able to rapidly process large datasets, predict pharmacokinetic profiles, and harness *in vivo* data to enhance compound design and prioritization. This innovation accelerates new drug development and paves the way for more personalized and potent therapeutic strategies.

In concert with this shift, Neal M. Davies’ Editorial “Technological Darwinism” [2] explores the adaptability of pharmaceutical sciences in the AI era, which is critical for the ongoing evolution of PK. Davies underscores the growing importance of AI in improving drug development workflows, a sentiment that resonates with our perspective on AI’s transformative role in pharmacokinetics. He advocates for an awareness of AI’s current limitations and promotes its ethical application, supplemented by human expertise. Such a collaborative approach is essential for the progression of personalized medicine and for ensuring the integrity and impartiality of the data that guide our decisions.

The traditional landscape of PK analysis has been dominated by software like WinNonlin, NONMEM, Monolix, and GastroPlus, which have been pivotal in predicting PK based on molecular properties. GastroPlus, for instance, has been acknowledged for its simulation capabilities in disease specific modeling that integrate physicochemical properties, biological data, and pharmacokinetic processes [3]. These tools have provided robust frameworks for the scientific community to understand and predict drug behavior within the body. However, the advent of AI-driven analysis promises to transcend these existing platforms by leveraging advanced algorithms to analyze complex datasets with greater speed, accuracy, and efficiency. AI has the potential not only to replicate but to significantly enhance the predictions of molecular behavior, offering deeper insights into drug absorption and metabolism, and thus opening new frontiers in personalized medicine. This evolution signifies a leap from static software models to dynamic AI systems that learn and adapt, fundamentally reshaping the PK domain.

## The AI revolution in pharmacology

Artificial Intelligence (AI) has revolutionized healthcare and pharmacology, as documented extensively by Maaike van der Lee et al. [4]. Their research sheds light on the diverse applications of AI in pharmacology research and clinical practice. According to the authors, AI and machine learning (ML) models have been creatively applied in pharmacology in a variety of contexts. Unsupervised clustering methods, for example, have demonstrated promise in locating putative medicinal substances and identifying patient groups who are well-suited for particular therapies. Supervised ML techniques have also proven useful in improving therapeutic medication monitoring. Drug levels in a patient’s system are analyzed to maximize therapeutic efficacy while minimizing side effects.

Natural language processing (NLP) is another important area where artificial intelligence has advanced significantly. NLP is increasingly being used to mine electronic health records for useful real world data. This method has enabled researchers to comprehend medication safety and efficacy profiles across a wide range of patient populations beyond the restricted scope of controlled clinical studies.

Maaike van der Lee et al.'s report highlights the transformative impact of AI on pharmaceutical practices and research. It particularly emphasizes AI’s ability to manage large datasets, learn, and adapt, which pave the way for novel advancements in patient care and drug development. AI’s roles range from predicting drug interactions to tailoring medication dosages for individual patients, demonstrating its extensive contributions to the field. This report underscores AI’s capacity to significantly enhance the efficiency and personalization of pharmaceutical services.

Furthermore, the integration of generative AI into pharmacokinetics marks a significant shift in healthcare, promising more effective and precisely tailored treatments. This advancement is not only about enhancing safety and efficacy; it is also reshaping the landscape of drug development and patient care into a more informed and patient-centric approach. Crucially, this AI-driven evolution in healthcare has the potential to reduce the number of subjects needed in clinical trials. By enabling more precise methodologies with higher efficacy, AI can minimize the required sample sizes, thereby significantly cutting costs and shortening the duration of trials. This aspect underscores the profound impact of AI in streamlining and optimizing pharmaceutical practices and research.

## AI-powered PK software–opportunities and applications

A recently published study [5] provides insight into how artificial intelligence and machine learning techniques are being used to transform the assessment of novel compounds’ drug metabolism and pharmacokinetics (DMPK) capabilities.

AI/ML techniques are in a unique position to leverage the vast amounts of data that are accessible in the biotech and pharmaceutical industries. This skill is especially important in the early stages of drug development when drug design and selection are greatly influenced by predictions of a compound’s behavior in the human body.

This predictive modeling can shed light on a compound’s absorption, distribution, metabolism, and excretion (ADME) properties, enabling initial evaluations without the need for extensive *in vitro* or *in vivo* research. These predictions are highly valuable in identifying which compounds to prioritize for further development, potentially saving considerable time and resources.

Moreover, PK software powered by generative AI opens the door to more individualized methods in medication development. These advanced techniques enable the analysis of large datasets to identify patient-specific characteristics that may influence drug metabolism. AI is also being used in PK to estimate the results of clinical studies, optimize dosage regimens, and anticipate possible drug-drug interactions.

To sum up, the integration of artificial intelligence into PK software signifies a noteworthy advancement in pharmaceutical research and development. AI serves as a powerful tool for comprehending and forecasting the behavior of novel pharmaceuticals, resulting in the development of safer and more efficient treatment options.

## Challenges and future directions

The integration of generative AI is significantly hampered by the dynamic nature of pharmacokinetic databases. The datasets that these artificial intelligence systems use can alter significantly as new studies are conducted and clinical trials are finished. AI needs to be able to recognize and adjust to inconsistencies that may arise from variations in study designs, patient demographics, and measurement methods, which can all contribute to variability in data quality. This is especially difficult when assessing the pharmacokinetic (DMPK) features of novel drugs and drug metabolism when precise and reliable data are crucial. AI needs to critically analyze and may need to reinterpret previous data in light of new results in addition to ingesting new data to remain effective. The AI systems require robust algorithms capable of managing these evolving datasets to ensure a comprehensive understanding of the ADME processes for both *in vitro* and *in vivo* systems [5].

Furthermore, Olga Obrezanova’s work [1] underscores the complexity of modeling pharmacokinetics in both animals and humans using AI. This process necessitates a deep understanding of the chemical and biological interactions at play, which can be challenging to encode within AI algorithms.

Envisioning the future of AI in pharmacokinetics, it’s ambitious yet conceivable to develop a comprehensive AI pharmacokinetics/pharmacometrics software. This advanced tool could potentially handle the entire spectrum of tasks from data cleaning and input optimization to model selection, improvisation of complex models, and performing exploratory data analysis (EDA), as well as both non-compartment (NCA) and compartmental analysis (CA). It could also conduct individual and population pharmacokinetics (popPK) studies, and generate complete reports adhering to FDA, EMA, or other regulatory guidelines. Such a tool, possibly integrating cutting-edge technologies like GPT, represents a significant leap in automating and enhancing pharmacokinetic research and analysis.

## Ethical considerations and transparency

Incorporating generative AI into pharmacokinetics necessitates thorough ethical consideration and transparency. As noted by Matthew R Wright [5], while AI offers significant opportunities for predicting pharmacokinetic behavior, it raises concerns about data adaptability, understanding of biological systems, and potential biases. Ethical guidelines are required to ensure that AI is used responsibly in predicting drug metabolism and pharmacokinetics. This includes ensuring data integrity, addressing biases in algorithms, and transparently reporting AI methodologies and limitations. As we advance in generative AI applications, it is crucial to foster an ethical framework that supports accurate, unbiased, and transparent pharmacokinetic research.

## Conclusion

The pharmaceutical sciences stand on the brink of an era redefined by AI, with the potential to revolutionize medication development towards highly personalized treatments. This technological leap promises to hasten and refine the drug development process, with AI-driven pharmacokinetics poised to transform precision medicine. However, this future raises critical questions about the ethical application of AI, the need for bias correction, and the maintenance of data integrity. As the scientific community navigates the intricacies of biological data and drug interactions, the development of robust ethical frameworks and transparent methodologies becomes crucial. The advancements in AI signal a future of dynamic, tailored, and effective healthcare, yet necessitate a cautious, collaborative, and multidisciplinary approach to ensure equitable benefits across society. With the right commitment, AI is set to fundamentally change drug discovery and pave the way for a new paradigm of individualized treatment, enhancing patient outcomes and expanding the pharmaceutical field.

## References

[1] Obrezanova O. Artificial intelligence for compound pharmacokinetics prediction. Curr Opin Struct Biol (2023) 79:102546. 10.1016/j.sbi.2023.102546 36804676

[2] Davies NM. Adapting artificial intelligence into the evolution of pharmaceutical sciences and publishing: technological darwinism. J Pharm Pharm Sci a Publ Can Soc Pharm Sci Societe canadienne des Sci pharmaceutiques (2023) 26:11349. 10.3389/jpps.2023.11349 PMC10075305 37034476

[3] Almukainzi M Jamali F Aghazadeh-Habashi A Löbenberg R. Disease specific modeling: simulation of the pharmacokinetics of meloxicam and ibuprofen in disease state vs healthy conditions. Eur J Pharmaceutics Biopharmaceutics (2016) 100:77–84. 10.1016/j.ejpb.2015.12.004 26752427

[4] van der Lee M Swen JJ. Artificial intelligence in pharmacology research and practice. Clin Translational Sci (2023) 16(1):31–6. 10.1111/cts.13431 PMC9841296 36181380

[5] Wright MR. Opportunities and considerations in the application of artificial intelligence to pharmacokinetic prediction. Methods Mol Biol (2022) 2390:461–82. 10.1007/978-1-0716-1787-8_21 34731483

